# Multifractal Behaviors of Stock Indices and Their Ability to Improve Forecasting in a Volatility Clustering Period

**DOI:** 10.3390/e23081018

**Published:** 2021-08-06

**Authors:** Shuwen Zhang, Wen Fang

**Affiliations:** Department of Finance, School of Economics and Management, Beijing Jiaotong University, Beijing 100044, China; 17241339@bjtu.edu.cn

**Keywords:** multifractal, forecasting, OSW-MF-DFA, GRU neural network, stock index time series

## Abstract

The financial market is a complex system, which has become more complicated due to the sudden impact of the COVID-19 pandemic in 2020. As a result there may be much higher degree of uncertainty and volatility clustering in stock markets. How does this “black swan” event affect the fractal behaviors of the stock market? How to improve the forecasting accuracy after that? Here we study the multifractal behaviors of 5-min time series of CSI300 and S&P500, which represents the two stock markets of China and United States. Using the Overlapped Sliding Window-based Multifractal Detrended Fluctuation Analysis (OSW-MF-DFA) method, we found that the two markets always have multifractal characteristics, and the degree of fractal intensified during the first panic period of pandemic. Based on the long and short-term memory which are described by fractal test results, we use the Gated Recurrent Unit (GRU) neural network model to forecast these indices. We found that during the large volatility clustering period, the prediction accuracy of the time series can be significantly improved by adding the time-varying Hurst index to the GRU neural network.

## 1. Introduction

In 1970, the economist Eugene F. Fama put forward the efficient market hypothesis (EMH), which became the cornerstone of contemporary financial theory. In this hypothesis, all information on the market will be quickly reflected in the stock price, so the stock prices are unpredictable [1]. However, later behavioral finance theory studies of market behavior have shown the limitations of the EMH. The criticisms focus on the irrationality of investors, market friction and incomplete arbitrage, which are in violation of the effective market hypothesis [2,3,4]. In empirical terms, momentum effects, reversal effects, January effects, and financial anomalies such as peaks and thick tails of time series, volatility clustering, etc. were also found in financial time series [5,6,7,8,9]. Hence, economists have actively sought new theories to explain these market anomalies. In 1994, Peters proposed the fractal market hypothesis (FMH). This hypothesis modifies the strict assumptions of the efficient market, pointing out that asset prices obey fractional Brownian motion, the return rate sequence has long memory, and the market may be in a non-equilibrium state [10]. Therefore, a certain level of predictability of prices has become a general consensus. After the FMH was proposed, the field mainly focused on two aspects, the study of the fractal characteristics of the stock market and building various models to try to predict market trends.

For the first aspect, regarding the method of studying fractal properties, the starting point is the rescaled range method (R/S) proposed by the British hydrologist Hurst [11]. When studying the relationship between the Nile Reservoir discharge and the water level, he found that a biased random walk (fractional Brownian motion) can well describe the long-term dependence of the two, so he proposed calculating the Hurst exponent by the rescaled range method, which was used for characterizing the self-similarity of time series. Many scholars have continuously optimized and improved the method. Peng et al., proposed the detrending fluctuation analysis method (DFA) when studying the long-range power-law correlation characteristics of DNA sequences, which became the mainstream for measuring the long-range correlation of stationary time series [12]. However, Kantelhardt et al., pointed out that in most cases, the scaling behavior of time series is very complicated and cannot be explained by a simple scaling index [13]. Therefore, the multifractal detrending volatility method (MF-DFA) was proposed and the author pointed out that a multifractal structure may come from the thick-tailed distribution and long-range correlation. Thompson et al., used multi-methods for analyzing the fractal characteristics of GE stock price series. The results showed that the MF-DFA model is better fitted [14]. A number of existing studies have shown that multifractals are common in financial markets in various countries, including stock markets [15,16,17,18,19], bonds [20] and Bitcoin markets [21]. The results above definitely all rejected the efficient market hypothesis. However, some scholars questioned the MF-DFA method. After comparing DFA, CMA, MF-DFA and other detrend volatility analysis methods, Bashan pointed out that the MF-DFA may result in false fluctuations, which may be reflected in the larger calculated generalized Hurst index [22]. This happens because the intervals divided by the MF-DFA method do not overlap, so the fitting polynomials of adjacent intervals may be discontinuous. Recognizing this shortcoming, many scholars use overlapping smoothing windows to optimize the model respectively, which reduces the spurious fluctuations caused by partially overlapping adjacent intervals [23,24]. We adopt this optimization method, which is called OSW-MF-DFA. Some scholars have studied the multifractal changes of the financial market under the impact of the pandemic and confirmed the reduction of market efficiency caused by COVID-19 [25,26,27]. Okorie studied the contagion effect of the fractal of the stock market, which proved the existence of multifractals from another aspect [28].

For the second aspect of predicting market trends, although stock markets are affected by various factors such as macroeconomic development, institutions, supervision, noise trading etc., researchers still try to construct various prediction models: from parametric models such as ARMA, ARIMA, and GARCH to machine learning such as BP, recurrent neural network (RNN), LSTM and GRU with gated structure, the prediction accuracy of the model has been continuously improved. The short-term memory neural network (LSTM) was proposed by Hochreiter and Schmidhuber in 1997 [29]. The gated recurrent networks LSTM and GRU, which have been popular in recent years, have been widely used to predict the trend of stock prices, and they have actually proved to have achieved good results by catching the long and short term memory of financial time series [30,31,32]. Yu et al., used the GARCH model and LSTM neural network to predict the volatility of China’s three major stock indexes, and the results proved that LSTM with long memory has better predictive ability [33]. However, with the popularization of LSTM, more and more studies have found that LSTM models have flaws such as limited explanatory power and slow convergence speed. Aiming at the shortcomings of LSTM, Cho et al., further optimized on the basis of LSTM and proposed a GRU neural network [34]. Compared with LSTM, GRU has only two gate control structures: update gate and reset gate, which reduces parameters while maintaining predictive performance, and it helps to speed up convergence [35,36].

In this article we aim to study the fractal properties of the Chinese and American intraday stock markets under the impact of the COVID-19 pandemic and use them to forecast by applying to the GRU model. According to the impact of the pandemic on the financial markets, we divide the time interval into three periods: before, during and after the first panic period of pandemic. In terms of multifractal research, this article utilizes the OSW-MF-DFA method optimized by overlapping smoothing windows. We obtain the generalized Hurst index and multifractal spectrum of the two stock indexes, then analyze and compare the fractal characteristics of the two markets at different periods. The time-varying Hurst exponent and its decomposition sequence are calculated by the DFA method, which are used as the input variables of the subsequent predictions. A time-varying Hurst sequence is also added to regular input variables such as opening price, closing price, highest price, lowest price, and volatility in GRU neural network, to explore whether it can improve forecasting efficiency.

Through empirical tests, we found that the two markets always have multifractal characteristics, and the degree of fractal intensifies during the pandemic. Among them, the US market was more affected by the pandemic. When putting time-varying Hurst and its decomposition sequence into the prediction model as inputs, we found that they can significantly improve the prediction performance of the model and outperform the volatility indicators during the panic period of pandemics (COVID-19) which has high volatility clustering.

## 2. Materials and Methods

### 2.1. Data

This article uses the CSI300 Index and the S&P 500 Index as representatives of the Chinese and US stock markets. The research sample is 5-min intraday data from 1 January 2019 to 9 June 2021. The CSI300 Index is China’s first cross-market index that reflects the overall Shanghai and Shenzhen markets. It consists of 300 mainstream stocks with the best market capitalization and liquidity in the Chinese stock market. It is the investment benchmark most valued by investors. Covering 500 high-quality companies on major exchanges such as the New York Stock Exchange and the Nasdaq, the S&P500 Index was established by the world’s authoritative rating agency Standard & Poor’s. It can reflect the entire picture of the US stock market. The initial indicators are the 5-min opening price, closing price, lowest price, and highest price. The data comes from the Wind financial terminal.

The COVID-19 pandemic in 2020 is a “black swan” event for the financial market, which has caused changes in the global financial environment. It is reasonable to speculate that the fractal situation of the market may change at different stages of the pandemic, and the features that help improve the prediction efficiency are also different. Considering that there are great differences between China and the United States in the time of the outbreak, it cannot be divided by a unified standard.

For the Chinese stock market, we adopt a pandemic development indicator constructed by Wang et al., who wrote a paper studying of the impact of the pandemic on the financial market [37]. They take into account the development of the pandemic and calculated the indicator to trace and forecast the trend of the pandemic. The study concluded that the number of new hospitalizations decreased to zero in early April. Therefore, this article divides the research interval of the CSI300 Index into the following three segments:

- Before the pandemic: 1 January 2019–31 December 2019
- Pandemic: 1 January 2020 (the starting point of the pandemic in China)–8 April 2020 (Wuhan unblocked)
- After the first panic period of pandemic: 9 April 2020–9 June 2021

For the US stock market, on the one hand, the rapidly developing pandemic has severely suppressed the market; on the other hand, the Federal Reserve and the Treasury Department have introduced unprecedented unlimited QE policies and large-amount rescue programs in their efforts to support the market. Cox found that the Fed’s policies had a significant impact on stock market behavior, and during the first panic period of pandemic, the market reflected more sentiment than substance [38]. Taking into account these two factors, this article divides the research interval of the S&P 500 index as follows:

- Before the pandemic: 1 January 2019–14 February 2020 (the starting point of the market plunge)
- Pandemic: 15 February 2020–16 June 2020 (the Fed shrinks its balance sheet for the first time, which means that the policy begins to tighten)
- After the first panic period of pandemic: 17 June 2020–9 June 2021

### 2.2. Descriptive Statistics

Before the multifractal analysis, perform descriptive statistics of the market’s return rate in the three stages, which can give us a whole picture of the market. At the same time, it is also a feasibility analysis for the follow-up empirical study. Considering it is unjustified to compare the results of two indices with the different levels directly, we make a mean normalization process for the sequence of returns before the study. The formula is as follows:

(1) $$r=\frac{r-\bar{r}}{r_{max}-r_{min}}$$

**Figure 1:**
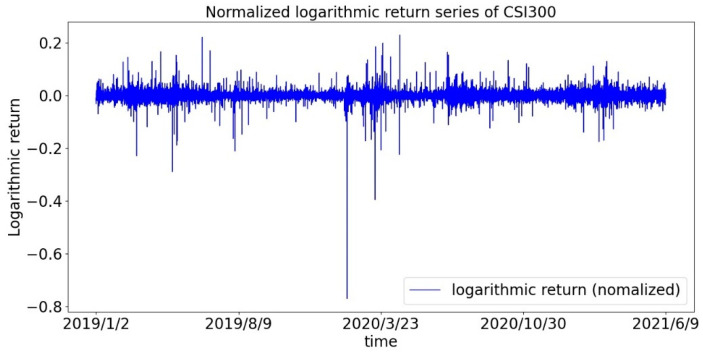
Time series of the logarithmic return of the CSI300 index.

**Figure 2:**
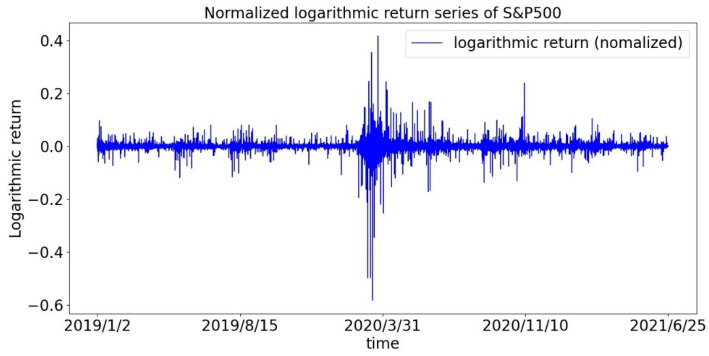
Time series of the logarithmic return of the S&P 500 index.

First, we draw the return sequences of the CSI300 Index and the S&P500 Index as shown in Figure 1 and Figure 2. It can be roughly seen that the return is not evenly distributed, but there is a phenomenon of volatility clustering, especially during the first panic period of the pandemic. Therefore, we assume that the sequence of returns does not obey a normal distribution.

Then, we calculate the statistical indicators of the return series. In addition to common indicators, such as mean value, extreme value, kurtosis and skewness, we also need to compare the degree of volatility clustering in each stage. As early as 1963, the French mathematician Mandelbrot proposed that the variance of financial asset prices has time-varying characteristics, and the variation range is clustered [39]. Then Engle first proposed Autoregressive Conditional Heteroskedasticity (ARCH) model to analyze the characteristics of price fluctuation [40]. Many scholars have confirmed the reliability of the model through empirical analysis [41,42,43]. In this paper, GARCH (1,1) model is used to measure volatility clustering. The model is as follows, and the sum of variable coefficients ($\beta_{1}+\delta_{1})$ in the variance equation is between 0 and 1. The closer it is to 1, the greater the degree of clustering:

(2) $$r_{t}=\alpha +u_{t}$$

(3) $$h_{t}=\beta_{0}+\beta_{1}{\left(u_{t-1}\right)}^{2}+\delta_{1}h_{t-1}$$

Table 1 shows the descriptive statistics of the two stock index markets in the three stages: before, during and after the first panic period of pandemic. The following conclusions are obtained through analysis:

- For the Chinese stock market, represented by the CSI300 Index, at each stage of the pandemic, the kurtosis of the return distribution is significantly greater than the standard value of the standard normal, indicating that the phenomenon of spikes and thick tails in the sequence always exists. Judging from the P-value of the Jarque-Bera test, the hypothesis of the standard normal distribution is strongly rejected, so, we concluded that the stock index market does not conform to the assumption of the traditional financial market, and it is necessary to study market efficiency from a fractal perspective. In addition, the pandemic has indeed had an impact on the market: (1) The coefficient of variation increased significantly during the pandemic, indicating the intensification of dispersion; (2) The skewness becomes smaller, indicating the degree of leftward deviation of the return distribution has increased; (3) kurtosis increases sharply, which indicates that the probability of extreme situations has increased, and the degree of thick tails has deepened; (4) The sum of GARCH (1,1) coefficients becomes larger, indicating that the volatility clustering effect becomes stronger. Above all, it is reasonable to assume that some features of the market have changed.
- For the US stock market, which is represented by the S&P500 Index, the spike and thick tail phenomenon and the impact of the pandemic are basically the same as the Chinese stock market, but with different degrees. There are two differences during the pandemic period: (1) The coefficient of variation of S&P 500 index increased faster and was much greater than that of CSI300 index; (2) The average return of the S&P500 index was positive, while that of the CSI300 index fell to negative.

In summary, descriptive statistics show us the market conditions at each stage of the pandemic. The most important conclusion is that there are spikes and thick tails and volatility clustering in the return series of the Chinese and American stock markets, which lays the foundation for the following fractal analysis.

### 2.3. Method of Testing Fractal: OSW-MF-DFA

The classic method of studying multifractal features is the multifractal detrended fluctuation analysis (MF-DFA) proposed by Kantelhardt in 2002 [13]. This method is extended by DFA and can be used to analyze the multifractal features of non-stationary time series. Its principle is to eliminate the local trend by dividing the sub-intervals, then fit the wave function of the residual series, and finally explore the power-law correlation of the wave function. We can obtain the generalized Hurst exponent to characterize the multifractal characteristics. However, the non-overlapping of the divided intervals may cause false fluctuations. Therefore, this paper adopts the overlapping smoothing window optimization method which has been verified by many scholars [23,24] and is called OSW-MF-DFA method. The specific steps are as follows:

(1) Suppose *R* (*i*) (*i* = 1, 2, …, *N*) is a certain time series, and *N* is the length of the series. First calculate the mean of the series, and construct the cumulative deviation series *Y*(*j*) as follows:
  (4) $$Y\left(j\right)=\overset{j}{\underset{i=1}{\sum }}(R\left(i\right)-\bar{R}),i=1\ldots N$$
(2) Divide *Y*(*j*) into $N_{s}=\left[\left(N-s\right)/\left(s-l\right)\right]$ subintervals with a unit length of *s*. The length of the overlapping part of adjacent intervals is *l*, which is the only improvement to the original method. Combined with previous experience, the value of *l* is usually s/3.
(3) For each subinterval, fit the univariate linear equation by the least square method. Then eliminate the local trend of each subinterval *v* (*v* = 1,2, … *N_s_*), and get the detrending residual sequence $Y_{v}\left(k\right)$ $p_{v}\left(k\right)$ is the local polynomial fitted value and we choose linear univariate polynomials:
  (5) $$y_{v}\left(k\right)=C_{0}+C_{1}vk+e_{k},\;k=1,2,\ldots ,s$$
  (6) $$Y_{v}\left(k\right)=y_{v}\left(k\right)-p_{v}\left(k\right),\;k=1,2,\ldots ,s$$
(4) Calculate the square mean and q-order volatility function of the residual sequence of *N_s_* subintervals, respectively:
  (7) $$F^{2}\left(s,v\right)=\frac{1}{s}\overset{s}{\underset{k=1}{\sum }}{\left(Y_{v}\left(k\right)\right)}^{2}$$
  (8) $$F_{q}\left(s\right)=\{\begin{array}{c} {\left\{\frac{1}{N_{s}}\overset{N_{s}}{\underset{v=1}{\sum }}{\left[F^{2}\left(s,v\right)\right]}^{\frac{q}{2}}\right\}}^{\frac{1}{q}},if\;q\neq 0\\ exp\{\frac{1}{2N_{s}}\overset{N_{s}}{\underset{v=1}{\sum }}ln\left[F^{2}\left(s,v\right)\right]\},if\;q=0 \end{array}$$
(5) Determine the scale index of the volatility function, and get the relationship between *F_q_* (*s*) and *s*. If *F_q_* (*s*) is a power-law distribution, the following formula is satisfied when q is constant:
  (9) $$F_{q}\left(s\right)\propto s^{h\left(q\right)}$$

The above formula is linearly fitted in the double logarithmic coordinate system, and the slope *h*(*q*) can be obtained, which is called the generalized Hurst exponent. It is used to describe the regional fractal changes of multifractals. Change the order *q*, repeat the above operation, observe the change of *h*(*q*):

(a) *h*(*q*) does not change with the increase or decrease of *q*, it is a single fractal system, otherwise it is multifractal.
(b) When *q* < 1, *h*(*q*) describes the fractal characteristics of small fluctuations; when *q* > 1, *h*(*q*) describes the fractal characteristics of large fluctuations; when *q* = 2, *h*(2) is the classical Hurst index, which measures long memory of the sequence as a whole.
(c) *h*(*q*) > 0.5 indicates that the sequence is persistent, 0 < *h*(*q*) < 0.5 indicates anti-persistence, *h*(*q*) = 0.5 indicates that the sequence is a random walk.

In addition to judging the multifractal by the change of the generalized Hurst index, the multifractal spectrum can also be drawn to further study the sensitivity of the time series to the large and small fluctuations. The multifractal spectrum characterizes the relationship between *f*(*α*) and α, and its calculation formula is as follows:

(10) $$\alpha =h\left(q\right)+q\;\frac{\partial h\left(q\right)}{\partial q}-\tau \;\left(q\right)$$

(11) τ (q)=qh(q)−1

(12) f (α)=qα−τ (q)

Among them, *α* is the local Hölder index, which is used to describe the degree of variation of the time series, so it is also called the singular index. τ (q) is called the Renyi index, which is another manifestation of the generalized Hurst index. *f*(*α*) is multifractal spectrum. The multifractal spectrum essentially reflects the same fractal characteristics as the generalized Hurst exponent, and has correspondence. The spectrum shape indicates the degree of fluctuation of the sequence. The wider the spectrum shape (that is the greater $\Delta \alpha =\alpha_{max}-\alpha_{min}$, the more intense the sequence fluctuation, the more uneven the internal distribution, and the greater the slope of the corresponding Hurst exponent with the order.

### 2.4. Price Prediction Model: GRU Neural Network

**Figure 3:**
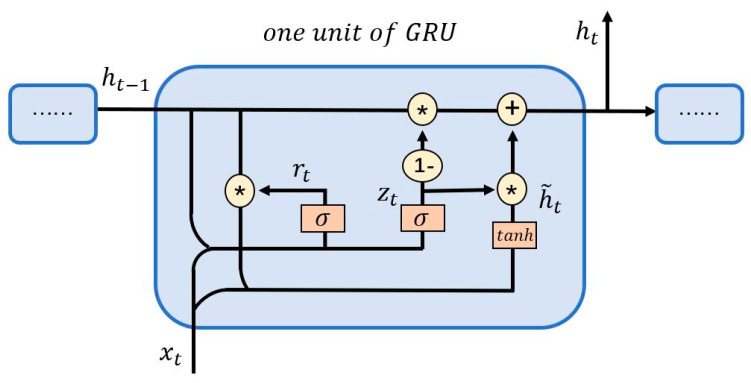
Unit structure diagram of GRU neural network.

Summarizing the research conclusions of other scholars, it is found that nonlinear neural networks have outstanding performance in prediction. In terms of predicting financial time series, the most popular ones are Long Short-term Memory Neural Network (LSTM) and Gated Recurrent Unit (GRU) [29,30,31,32,33,34,35]. The latter was proposed by Cho et al., in 2014 and it is a variant of LSTM [34]. The main change is that the “cell state” that transmits information is removed, but it can also achieve the effect of transmitting long-term memory. This paper selects GRU as the prediction model, and its unit structure diagram is shown in Figure 3.

In Figure 3, *x_t_* is the input value at the current moment. $h_{t-1}$ is the output at the previous moment. ${\tilde{h}}_{t}$ is the hidden state at the current moment. *h_t_* is the output at the current moment. *r_t_* and *z_t_* are the reset gate and the update gate respectively, and the former determines how much of the hidden state $h_{t-1}$ at the previous moment needs to be forgotten, the latter controls the extent to which the information of the previous state is passed into the current state. tanh is the activation function to ensure that the output result is between −1 and 1. *W* and *U* are the weights of information transmitted between each node. The relationships between the variables are as follows:

(13) $$z_{t}=\sigma \left(W_{z}x_{t}+U_{z}h_{t-1}\right)$$

(14) $$r_{t}=\sigma \left(W_{r}x_{t}+U_{r}h_{t-1}\right)$$

(15) $${\tilde{h}}_{t}=\tanh\left[Wx_{t}+U\left(r_{t}*h_{t-1}\right)\right]$$

(16) $$h_{t}=\left(1-z_{t}\right)*h_{t-1}+z_{t}*{\tilde{h}}_{t}$$

## 3. Results

### 3.1. Multifractal Test

The multifractal testing method in this paper adopts OSW-MF-DFA and the procedure is based on the research of Espen [44]. We test the multifractal behavior of 5-min’s logarithmic return series.

Firstly, we draw the curves of the generalized Hurst index with order q, and the range of q is (−10,10), which is used to measure degrees of volatility. The situations of the two markets are as follows.

**Figure 4:**
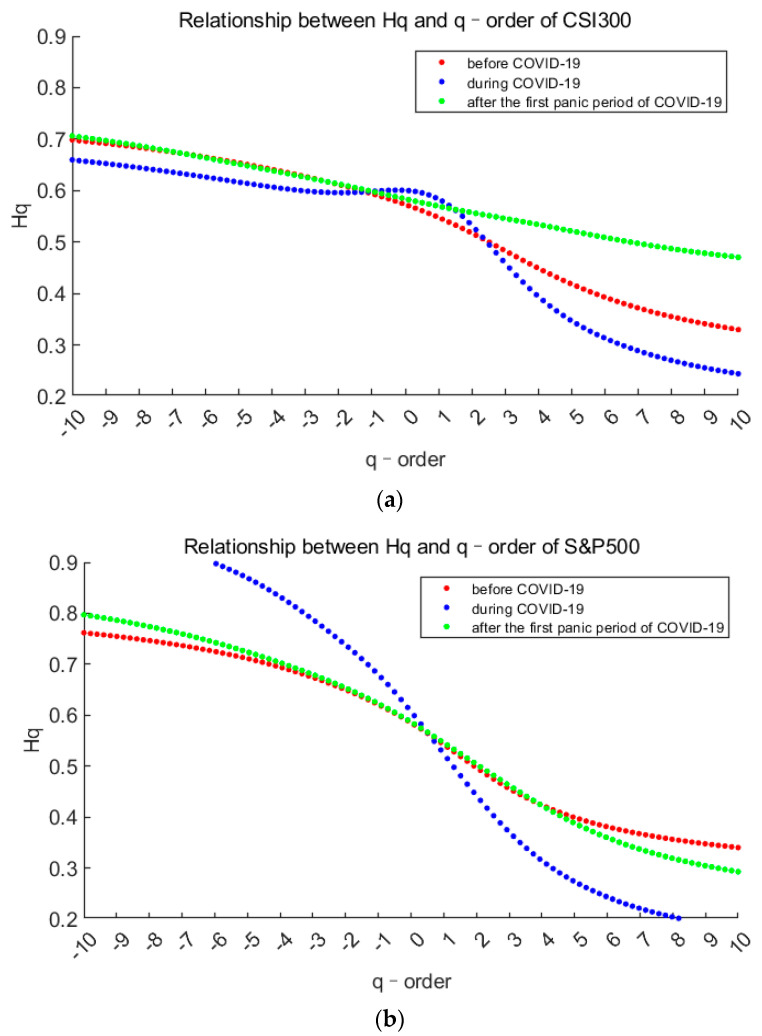
The generalized Hurst index varies with the order q. (**a**,**b**) stands for CSI300 index and S&P500 index respectively. Red represents before the pandemic, blue represents during the first panic period of pandemic, and green represents after the first panic period of pandemic.

It can be observed from the Figure 4 that, there are two things in common for all three stages of the two markets. On one hand, the generalized Hurst indices decrease as the order *q* increases, which shows that the market always have multifractal characteristics. On the other hand, there are obvious differences in the curves of the three stages, indicating that the degree of fractal is not the same. In more detail, the Hurst curves before and after the first panic period of pandemic are similar in shape. Especially when *q* < 1, the Hurst value of each market is greater than 0.5, indicating that small fluctuations are persistent before and after the first panic period of pandemic. But the curves change greatly of both markets during first panic period of the pandemic. In terms of CSI300 index, when *q* > 2, the Hurst index during the pandemic quickly fell below 0.5 and became the minimum one, which shows that super large fluctuations have a strong anti-persistence. In terms of S&P500 index, Hurst curves during the pandemic are in a reverse “S” shape, indicating that small fluctuations are strongly persistent, while large fluctuations are strongly anti-persistent. From the changes in the *Hq* as *q* > 4 of the S&P500 index before and after the panic period, we find that large fluctuations have greater anti-persistence after the panic, but it’s the opposite in the Chinese market. Which mean it is stronger risk aversion in S&P500 stock market.

Through the above Hurst curve, it is easier for us to compare the changes of a single market in three stages. Next, by analyzing the multifractal spectrum and its parameters, we can compare two markets better.

**Figure 5:**
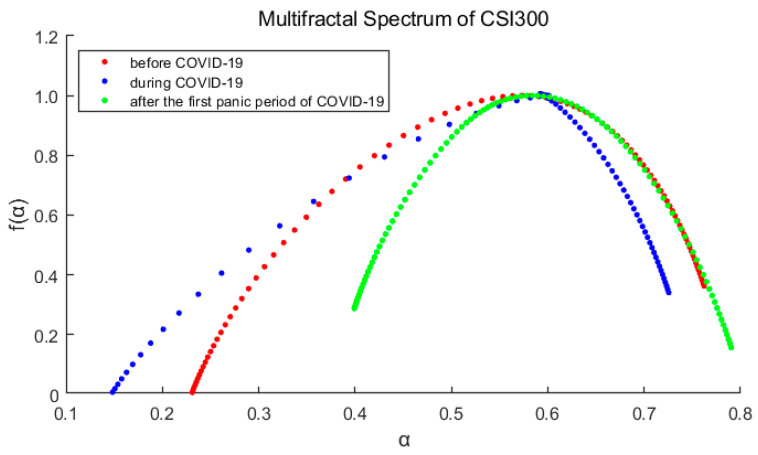
Multifractal spectrum of the CSI300 Index varies with the order *q*. Red represents before the pandemic, blue represents during the pandemic, and green represents after the first panic period of pandemic.

**Figure 6:**
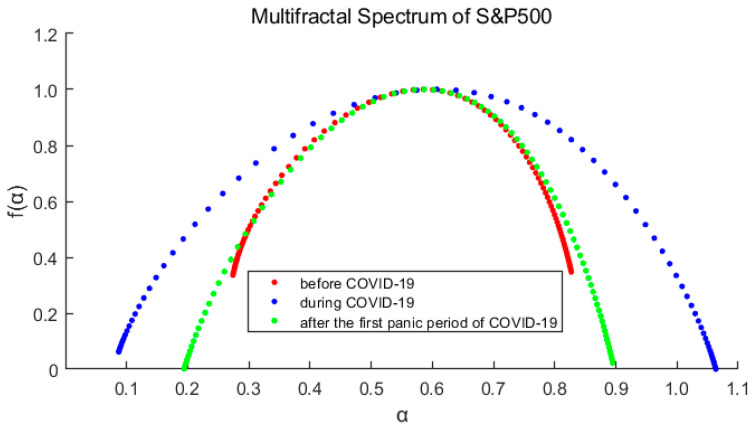
Multifractal spectrum of the S&P500 Index varies with the order *q*. Red represents before the pandemic, blue represents during the pandemic, and green represents after the first panic period of pandemic.

Figure 5 and Figure 6 show the multifractal spectra of each stage of the markets. As we can see in each figure, the three spectrum lines are single-peaked, which verifies the conclusion that the market has multifractal characteristics. At the same time, it is also observed that the spectrum shape and width are obviously different.

The main parameters for calculating the fractal spectrum are obtained from the Table 2 and Table 3. The greater Δα ($=\alpha_{max}-\alpha_{min}$) indicates the deeper degree of multifractal. Δf ($=f\left(\alpha_{max}\right)-f\left(\alpha_{min}\right)$) measures the relative probability of the maximum and minimum of the rate of return. If Δf>0, it means that the probability of the maximum rate of return is greater, otherwise the minimum is more likely. In this paper, we define the skewness index R($=\Delta \alpha_{r}-\Delta \alpha_{l}$) to measure the dominance of the larger and smaller rates of return. If *R* > 0, the larger rate of return is more advantageous.

After comparing the spectrums and their parameters, we can reach three main conclusions:

- For each market, ∆*α* is the largest of the three periods during the pandemic, indicates that the pandemic has intensified the multifractal degree of the market. And ∆*α* of S&P500 is correspondingly larger than that of CSI300, which means the higher fractal degrees of US stock market (it also means more inefficient).
- The spectrum shape of S&P500 is more symmetrical with Δf and *R* closing to 0, while those of CSI300 indicate an obvious tailing phenomenon. It shows the time variability and imbalance of large and small returns in China’s stock market. Especially after the first panic period of pandemic, the long right tail of CSI300 should be paid more attention, which indicating that the proportion of large yields has increased and occupied a dominant position.
- From the above analysis, we find that the US market was more deeply impacted by the pandemic. After the first panic period of pandemic, the fractal degrees of both markets have partially recovered, which indicates that the impacts of the pandemic on the markets have not subsided, although the indices have rebounded.

### 3.2. Time-Varying Hurst Sequence

The fractal situation of the market return rate reflects the fluctuation characteristics of the price, and it is expected to improve the accuracy of the forecast model as an input feature. Multifractal originates from long-range correlation and thick-tailed distribution, the latter can be eliminated by shuffling the sequence. This article assumes that long-range correlation is more helpful for price prediction. Therefore, the overall Hurst index and $Hurst_{corr}$ caused by long-range correlation are calculated as follows:

(17) $$Hurst_{corr}=Hurst-Hurst_{shuffle}$$

**Figure 7:**
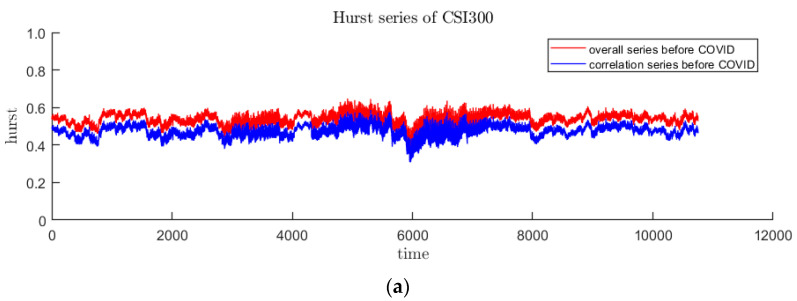

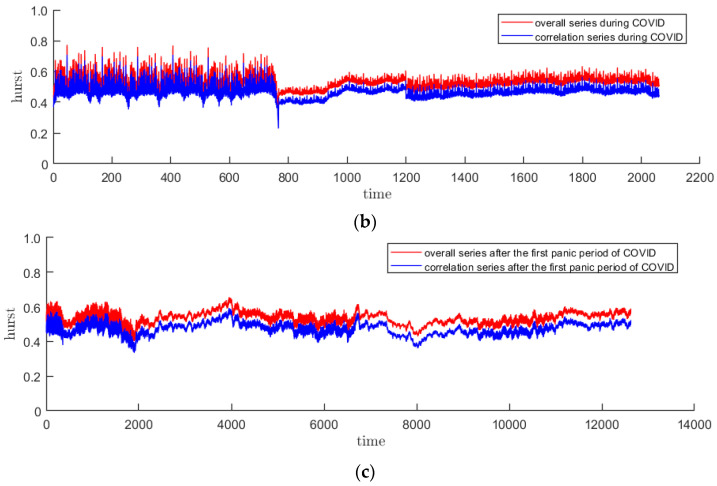
The red curve represents the overall Hurst of CSI300, and the blue curve represents $Hurst_{corr}$ caused by long-range correlation. (**a**–**c**) are before, during and after the first panic period of pandemic, respectively.

**Figure 8:**
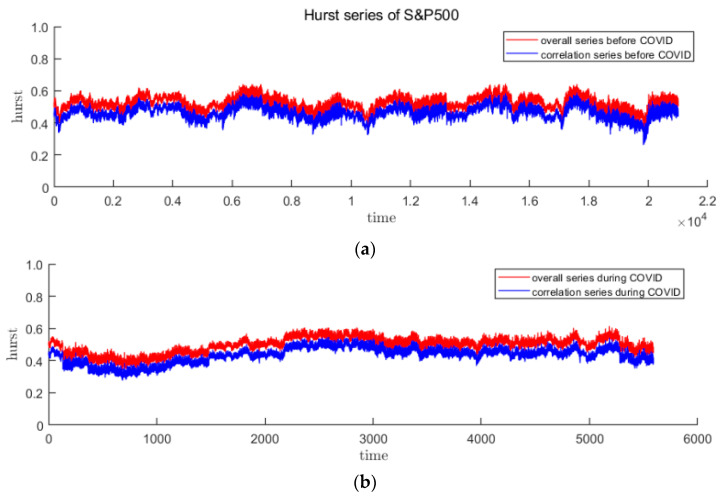

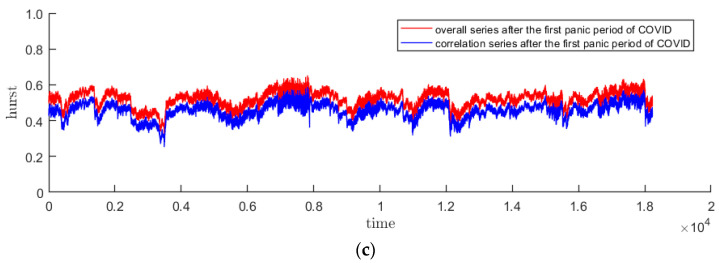
The red curve represents the overall Hurst of S&P500, and the blue curve represents $Hurst_{corr}$ caused by long-range correlation. (**a**–**c**) are before, during and after the first panic period of pandemic, respectively.

In this paper, the DFA method with sliding window is used to calculate the time-varying Hurst index sequence. DFA is used to measure the single fractal characteristics of the sequence over a period of time. Since the time of the sliding window will not be too long, the fractal situation of the samples in the window will not change too much. It is reasonable to use this method. Drawing on the research experience of other scholars, the sliding window is selected as 20 trading days, and the step size is 1. The time-varying Hurst index sequences of the three stages of CSI300 and S&P500 are obtained as Figure 7 and Figure 8. It is easy to find that in the pre- and post-pandemic stages, the time-varying Hurst index roughly falls between 0.4 and 0.6, indicating that the trend or counter-trend of the sequence is small, while the fluctuations during the pandemic have increased significantly and the fractal status is complex. In addition, it can be seen from the decomposition that long-range correlation is the main cause of multifractal, because $Hurst_{corr}$ curve lies right below the Hurst curve.

### 3.3. Price Forecast Analysis

On the basis of fractal research, we further explore its application in price prediction. This paper uses the GRU neural network that performs well in the financial field, combined with fractal features and other variables to construct price prediction model. Next, I will focus on five problems: (1) the division of data sets; (2) the selection of optimal parameters; (3) the selection of inputs; (4) the comparison of the pros and cons of models; (5) the analysis of prediction results.

#### 3.3.1. Divide the Data Sets

In terms of data set division, this article inherits the “80–20%” rule which is widely used in kinds of scientific research [45,46]. That is, the first 80% of each interval is divided into the training set for optimal model training, and the last 20% is the test set to test the prediction performance. Although using sliding windows to calculate Hurst index and volatility requires some loss of data, the total number of samples is large enough to not affect the effect of model training.

#### 3.3.2. Set Parameters

In the case of a given model structure, the main factors that affect the prediction effect are parameter values and input variables. The optimal parameters are determined through multiple attempts, and the input variables are selected by logical analysis. The parameters to be adjusted in this article are as follows:

History, that is, how long in the past data is used for prediction. Considering that the price series is memorable, too short historical values may cause some information to be lost, but too long historical values may also contain redundant information. In the case of controlling other parameters unchanged, the sensitivity analysis of the historical value found that it has a “U”-shaped relationship with the model error. And the error is the smallest when the value is set to 20.

Units, that is, the number of unit nodes in the hidden layer, determines the dimension of the data transferred to the next layer. The more the number of nodes, the more the number of parameters to be fitted, and the longer the time required for model training. After many attempts to change the number of units, it was found that this parameter has an irregular relationship with the error. Finally, combining the experience of other scholars, we tried between (10, 40) and determined that the optimal result was reached when units = 12.

The Optimizer is an algorithm that minimizes the loss function of the neural network when fitting parameters, which is of great significance to the effect of machine learning. In the pursuit of speed and accuracy, the optimization algorithm is constantly updated and iterative, from the early simple but inefficient stochastic gradient descent (SGD) to Adagrad, which efficiently distinguishes high and low frequency parameters. At present, the most widely used optimizer is Adam. After testing, Adam’s optimization effect is the best in all cases.

In addition to the above three most important parameters, there are also learning rounds (epoch), batch size (batch size), activation function (activation), etc. We use the same method to find the optimal value of each parameter in turn, and finally determines Epoch = 500, Batch Size =100, and the activation function is Relu.

The optimal parameters are summarized in Table 4.

#### 3.3.3. Select Input Variables

It has been mentioned that the price of an asset can be divided into volatility and trend, so these two aspects of information should be considered when selecting characteristics. Generally speaking, the general market indicators of the index basically contain trend and volatility information, including opening price, closing price, highest price, and lowest price. For example, the closing price at the previous moment is the reference for the opening price at the next moment, reflecting the trend, and the spread between the opening and closing prices also reflects fluctuations. In addition, volatility indicators such as the rate of change in return and volatility can be easily calculated through the price. On this basis, this paper innovatively adds two other volatility indicators: time-varying Hurst and $Hurst_{corr}$.

#### 3.3.4. Forecast of CSI300 Index

After determining the parameters and variables to be input, this paper uses the GRU neural network to predict the 5-min intraday prices of the two indices. The model is built based on Python 3.6.4 with function libraries including keras, tensorflow, pandas, numpy, matplotlib, etc. Among them, keras and tensorflow are used to build the main body of GRU; pandas and numpy are used for data reading and writing; matplotlib is used to draw prediction results graphs. The evaluation indicator of the model is the loss function. This article selects the classic mean absolute error (MAE) and root mean square error (RMSE). The calculation formulas for the two are as follows:

(18) $$MAE=1/n\overset{n}{\underset{i=1}{\sum }}|h(x_{i})-y_{i}|$$

(19) $$RMSE={\left\{1/n\overset{n}{\underset{i=1}{\sum }}(h(x_{i})-y_{i})\right\}}^{\frac{1}{2}}$$

The purpose of prediction is to explore whether the time-varying Hurst index can improve the prediction effect of the model whether it is better than traditional volatility indicators, especially in the stage of volatility clustering.

Based on the above objectives, the combination of input variables is designed as follows. Table 5 shows the prediction results.

- Num 1: closing price, opening price, highest price, lowest price, volatility
- Num 2: closing price, opening price, highest price, lowest price, *Hurst*
- Num 3: closing price, opening price, highest price, lowest price, $Hurst_{corr}$

**Figure 9:**
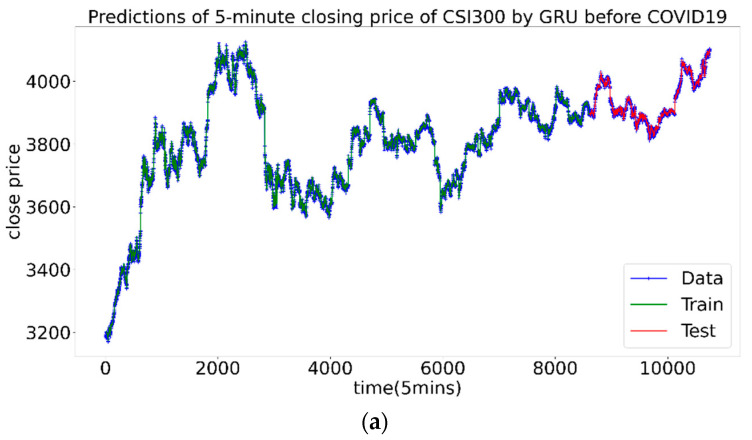

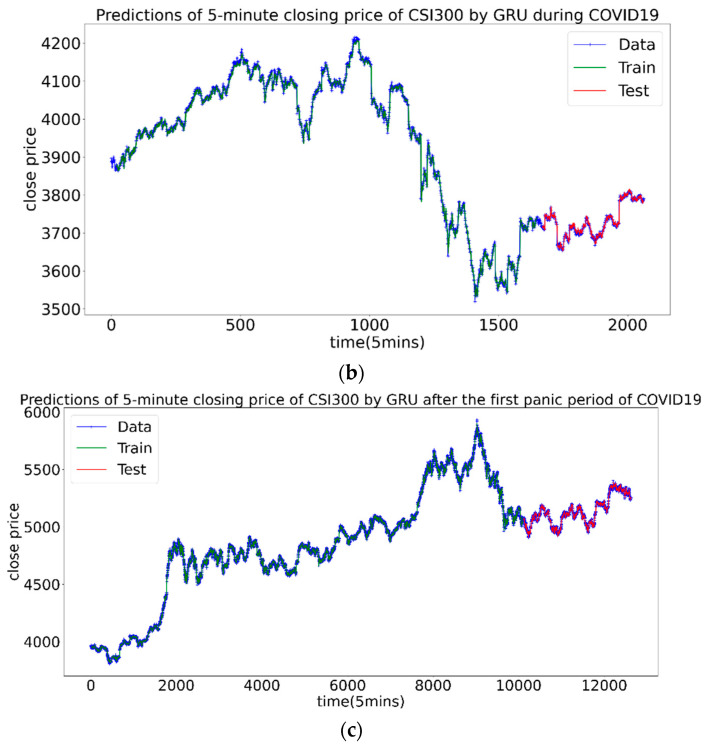
The prediction results of the CSI300 Index, the green part is the training set, and the red part is the test set. (**a**–**c**) are before, during and after the first panic period of pandemic, respectively.

It can be seen from the results that at different stages, the best combination is not the same. Before the pandemic, the prediction errors of three combinations were basically close, Num 2 with the Hurst index won out with a slight advantage. During the pandemic, Hurst and $Hurst_{corr}$ outperformed the volatility rate. The best one is Num 3 with the $Hurst_{corr}$, of which the MAE and RMSE of the test set were significantly smaller than the other two, indicating that at this stage $Hurst_{corr}$ contains the most effective information. After the first panic period of pandemic, the prediction effects of the three combinations were close, and the Num 1 with volatility was slightly dominant. Figure 9 shows the prediction diagrams of price by neural network.

Three conclusions can be drawn: First, in the period of large volatility clustering, the time-varying Hurst index can indeed improve the prediction accuracy. $Hurst_{corr}$ caused by the long-range correlation performed best. Second, in the period when the volatility clustering effect is relatively not obvious or dispersion is small, the volatility, Hurst and $Hurst_{corr}$ contribute almost the same to the forecast. Third, during the pandemic, the prediction effect is the worst. This is consistent with common sense, that is, the greater the market volatility, the more difficult it is to predict. For the prediction of the CSI300 Index, the assumption that the Hurst Index can improve the forecasting efficiency has been proved to be correct.

#### 3.3.5. Forecast of S&P500 Index

Similarly, applying the above three combinations to the prediction model of S&P500, the prediction errors at each stage are shown in Table 6.

**Figure 10:**
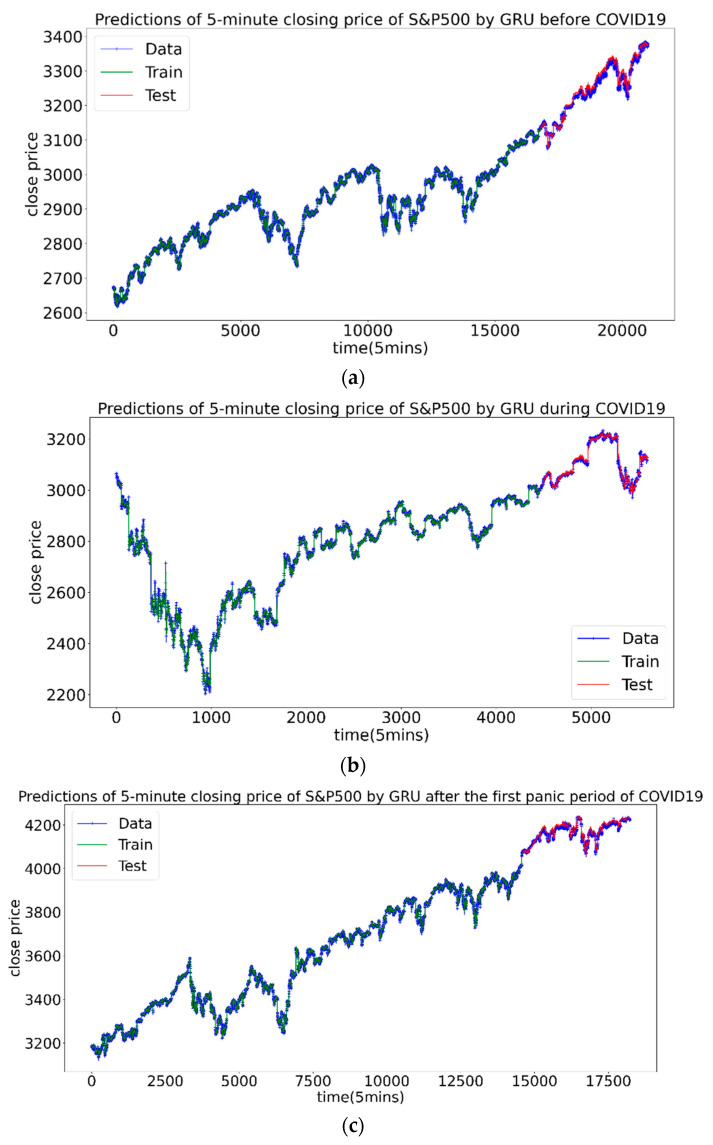
The prediction results of the S&P500 Index, the green part is the training set, and the red part is the test set. (**a**–**c**) are before, during and after the first panic period of pandemic, respectively.

The analysis shows that one thing is consistent with the conclusion of CSI300: during the pandemic, adding $Hurst_{corr}$ can significantly improve the forecasting effect, and outperforms the volatility and the overall Hurst index. In addition, there are two different conclusions: First, for the S&P500 index, before and after the first panic period of pandemic, the volatility index has obvious advantages in forecasting. Second, comparing the minimum forecast errors of the three stages, the results are generally larger than the corresponding CSI300 forecast errors. This shows that the US market is more difficult to predict. There may be differences in the characteristics of the Chinese and US stock markets, and more new indicators are needed to characterize them. Figure 10 shows the prediction diagrams of price by neural network.

## 4. Conclusions and Discussion

In this paper we take CSI300 and S&P500 as examples to study the fractal properties of 5 min stock indices under the impact of the pandemic and add the fractal features to the GRU neural network for price prediction. The research period is from 1 January 2019 to 9 June 2021. According to the development of the pandemic, it is further divided into three stages before, during and after the first panic period of pandemic. Our study has the following important conclusions: In terms of fractal research, we use OSW-MF-DFA to test the multifractal properties of the market return time series. We find that: (1) Multifractals are always present in all three stages of the two markets, but there are differences in the fractal characteristics of different markets and different stages. Our results show that the multifractals of the two markets have increased significantly during the pandemic, and the US market has been more affected by the pandemic. (2) After the first panic period of pandemic, the fractal degrees of two markets have declined, but they are still higher than the ones before the pandemic. In terms of price prediction, we set three sets of inputs to study the prediction effect of Hurst index and $Hurst_{corr}$ compared to volatility. The research found that: (1) For CSI300 and S&P500, $Hurst_{corr}$ can significantly reduce the forecast error during the large volatility clustering period (during the pandemic), especially for the S&P500 market. (2) The forecast error of S&P500 is significantly greater than that of CSI300, indicating that there are differences in the characteristics of the Chinese and American stock markets.

This study also has some suggestions for market agents. For market participants, it provides a way to judge the current market efficiency through fractal analysis. This study shows that Hurst index may be a good timing index and adding fractals can make predictions more accurate during the panic period of market. For policy makers, it has become a new method to study the intervention degree of fiscal and monetary policy on the market through fractal situation. For regulators, such as exchanges and CSRC, they can supervise the operation of the current market from the perspective of multifractal. When the efficiency of the market is reduced due to a major impact such as the pandemic, the trading rules could be modified appropriately to improve the stability of the market.

Our work is a study on the fractal and forecast of the market under the impact of the pandemic. By adding Hurst index as an input element in the GRU neural network, we improved the forecast accuracy during the panic period. But before and after that it is not contribute to the prediction accuracy, so the future research can join a dynamic system to determine whether to add the Hurst index when forecasting.

## Figures and Tables

**Table 1:** Specific descriptive statistics of CSI300 and S&P500 in three stages.

Stage	Before the Pandemic		During the Pandemic		After the First Panic Period	
market	CSI300	S&P500	CSI300	S&P500	CSI300	S&P500
Mean	−2.50 × 10^−19^	−4.11 × 10^−19^	−4.75 × 10^−19^	7.22 × 10^−19^	2.40 × 10^−18^	4.48 × 10^−19^
Max	0.43313	0.40504	0.20563	0.41758	0.48440	0.63640
Min	−0.56687	−0.59450	−0.794374	−0.58241	−0.51560	−0.36360
C.V. ^1^	81.00000	84.00000	247.00000	380.00000	84.50000	57.50000
Skewness	−0.912033	−0.942465	−11.88979	−3.37300	0.06833	1.38635
Kurtosis	49.79279	70.36161	365.45870	123.23470	16.13560	78.50269
*p*-value ^2^	0.00000 ***	0.00000 ***	0.00000 ***	0.00000 ***	0.00000 ***	0.00000 ***
GARCH (1,1) ^3^	0.98075 ***	0.91218 ***	1.00000 ***	0.96622 ***	0.97298 ***	0.88691 ***

^1^ C. V. represents the coefficient of variation, which is the ratio of standard deviation to mean value. It is used to compare the dispersion of two sets of data with different scales. ^2^ It is the *p*-value of Jarque-Bera test, which is used to judge the normal distribution. ^3^ GARCH (1,1) represents the sum of coefficients in the GRACH (1,1) model. The value falls between the (0,1), which is used to measure the degree of fluctuation aggregation. The closer it is to 1, the higher the degree of volatility clustering. *** Significant at the confidence level of 1%.

**Table 2:** Detailed parameter values of CSI300 multifractal spectrum.

Stage	$\alpha_{min}$	$\alpha_{max}$	Δα	$f\left(\alpha_{min}\right)$	$f\left(\alpha_{max}\right)$	Δf	R
before	0.2214	0.7490	0.5276	−0.0599	0.3761	0.4360	−0.1852
during	0.1433	0.7210	0.5777	−0.0524	0.3508	0.4032	−0.3213
after	0.4186	0.7799	0.3613	0.3628	0.1756	−0.1872	0.0317

**Table 3:** Detailed parameter values of S&P500 multifractal spectrum.

Stage	$\alpha_{min}$	$\alpha_{max}$	Δα	$f\left(\alpha_{min}\right)$	$f\left(\alpha_{max}\right)$	Δf	R
before	0.2738	0.8271	0.5532	0.3364	0.3474	0.0110	−0.0719
during	0.0870	1.0660	0.9792	0.0615	−0.0248	−0.0863	−0.0610
after	0.1863	0.8948	0.7084	−0.0634	0.0228	0.0862	−0.0917

**Table 4:** Detailed parameter values of S&P500 multifractal spectrum.

Parameters	History	Units	Optimizer	Epoch	Batch Size	Activation
value	20	12	Adam	500	100	Relu

**Table 5:** The prediction results of the CSI300 Index at each stage. The black italic font indicates the best combination for each stage.

Stage	Num	MAE-Train	RMSE-Train	MAE-Test	RMSE-Test
before	1	4.74	7.22	3.13	4.29
	***2***	***4.67***	***7.06***	***3.11***	***4.23***
	3	4.68	7.08	3.13	4.25
during	1	6.42	12.20	8.86	12.7
	2	7.12	11.08	5.18	11.08
	***3***	***7.36***	***11.25***	***4.96***	***7.50***
after	***1***	***6.77***	***9.57***	***5.76***	***7.84***
	2	7.01	9.83	6.02	8.13
	3	6.96	9.78	5.94	8.04

**Table 6:** The prediction results of the S&P500 Index at each stage. The black italic font indicates the best combination for each stage.

Stage	Num	MAE-Train	RMSE-Train	MAE-Test	RMSE-Test
before	***1***	***1.51***	***2.60***	***8.36***	***9.85***
	2	1.65	2.69	11.55	13.68
	3	1.61	2.66	12.39	14.69
during	1	5.94	11.05	18.13	22.76
	2	6.98	11.88	8.32	11.56
	***3***	***6.94***	***11.89***	***6.13***	***9.55***
after	***1***	***3.00***	***4.72***	***7.10***	***8.43***
	2	2.73	4.49	19.49	21.04
	3	2.72	4.48	24.28	25.76

## Data Availability

Publicly available datasets were analyzed in this study. This data can be found here: https://github.com/Garfield0127/multifractal.

## References

[1] Fama E.F. (1970). Efficient capital markets: A review of theory and empirical Work. J. Financ..

[2] Daniel K., Titman S. (1999). Market efficiency in an irrational world. Financ Anal. J..

[3] Hou K., Moskowitz T.J. (2005). Market frictions, price delay, and the cross-section of expected returns. Rev. Financ. Stud..

[4] Lee S., El Meslmani N., Switzer L.N. (2020). Pricing Efficiency and Arbitrage in the Bitcoin Spot and Futures Markets. Res. Int. Bus. Finance.

[5] Bos J.W.D. (1994). Stock market efficiency the evidence from FTA indices of eleven major stock markets. De Econ..

[6] Van Der Sar N.L. (2003). Calendar effects on the Amsterdam stock exchange. De Econ..

[7] Bae H.-O., Ha S.-Y., Kim Y., Lim Y., Yoo J. (2020). Volatility flocking by cucker–smale mechanism in financial markets. Asia-Pac. Financ. Mark..

[8] Moskowitz T.J., Grinblatt M. (1999). Do industries explain momentum?. J. Financ..

[9] Louis H. (2004). Earnings management and the market performance of acquiring firms. J. Financ. Econ..

[10] Peters E. (1994). Fractal Market Analysis. Applying Chaos Theory to Investment and Analysis.

[11] Hurst H.E. (1951). Long term storage capacity of reservoirs. Trans. Am. Soc. Civ. Eng..

[12] Peng C.-K., Buldyrev S., Havlin S., Simons M., Stanley H.E., Goldberger A.L. (1994). Mosaic organization of DNA nucleotides. Phys. Rev. E.

[13] Kantelhardt J.W., Zschiegner S.A., Koscielny-Bunde E., Havlin S., Bunde A., Stanley H.E. (2002). Multifractal detrended fluctuation analysis of nonstationary time series. Phys. A Stat. Mech. Its Appl..

[14] Thompson J.R., Wilson J.R. (2016). Multifractal detrended fluctuation analysis: Practical applications to financial time series. Math. Comput. Simul..

[15] Fang W., Wang J. (2012). Statistical properties and multifractal behaviors of market returns by ising dynamic systems. Int. J. Mod. Phys. C.

[16] Pascoal R., Monteiro A.M. (2014). Market efficiency, roughness and long memory in PSI20 index returns: Wavelet and entropy analysis. Entropy.

[17] Han C.Y., Wang Y.M., Ning Y. (2019). Analysis and comparison of the multifractality and efficiency of Chinese stock market: Evidence from dynamics of major indexes in different boards. Phys. A Stat. Mech. Its Appl..

[18] Tiwari A.K., Aye G.C., Gupta R. (2019). Stock market efficiency analysis using long spans of Data: A multifractal detrended fluctuation approach. Financ. Res. Lett..

[19] Ge X., Lin A. (2021). Multiscale multifractal detrended partial cross-correlation analysis of Chinese and American stock markets. Chaos Solitons Fractals.

[20] Wang H.Y., Wang T.T. (2018). Multifractal analysis of the Chinese stock, bond and fund markets. Phys. A Stat. Mech. Appl..

[21] Fang W., Tian S., Wang J. (2018). Multiscale fluctuations and complexity synchronization of Bitcoin in China and US markets. Phys. A Stat. Mech. Appl..

[22] Bashan A., Bartsch R., Kantelhardt J.W., Havlin S. (2008). Comparison of detrending methods for fluctuation analysis. Phys. A Stat. Mech. Appl..

[23] Tang Y., Zhu P.F. (2019). Research of long memory, risk and efficiency of bull and bear based on CSI300 index futures: From the perspective of multifractality. Mange. Rev..

[24] Rak R., Zieba P. (2015). Multifractal flexibly detrended fluctuation analysis. Acta Phys. Pol. B.

[25] Aslam F., Mohti W., Ferreira P. (2020). Evidence of intraday multifractality in european stock markets during the recent Coronavirus (COVID-19) outbreak. Int. J. Financial Stud..

[26] Naeem M.A., Farid S., Ferrer R., Shahzad S.J.H. (2021). Comparative efficiency of green and conventional bonds pre- and during COVID-19: An asymmetric multifractal detrended fluctuation analysis. Energy Policy.

[27] Mnif E., Jarboui A. (2021). COVID-19, bitcoin market efficiency, herd behavior. Rev. Behav. Financ..

[28] Okorie D.I., Lin B. (2021). Stock markets and the COVID-19 fractal contagion effects. Financ. Res. Lett..

[29] Hochreiter S., Schmidhuber J. (1997). Long short-term memory. Neural. Comput..

[30] Fischer T., Krauss C. (2018). Deep learning with long short-term memory networks for financial market predictions. Eur. J. Oper. Res..

[31] Wu D., Wang X., Su J., Tang B., Wu S. (2020). A Labeling method for financial time series prediction based on trends. Entropy.

[32] Nabipour M., Nayyeri P., Jabani H., Mosavi A., Salwana E., Shahab S. (2020). Deep learning for stock market prediction. Entropy.

[33] Yu S.L., Li Z., Wang R., Chen Z. (2019). Forecasting Stock Price Index Volatility with LSTM Deep Neural Network. Proceedings of the 11th International Conference on Modelling, Identification and Control.

[34] Cho K., Van Merriënboer B., Gulcehre C., Bahdanau D., Bougares F., Schwenk H., Bengio Y. Learning phrase representations using RNN encoder-decoder for statistical machine translation. Proceedings of the Conference on Empirical Methods in Natural Language Processing (EMNLP 2014).

[35] Luo L.-X. (2018). Network text sentiment analysis method combining LDA text representation and GRU-CNN. Pers. Ubiquitous Comput..

[36] Zhao J., Zeng D., Liang S., Kang H., Liu Q. (2021). Prediction model for stock price trend based on recurrent neural network. J. Ambient. Intell. Humaniz. Comput..

[37] Wang H., Zhang Y., Lu S., Wang S. (2020). Tracking and forecasting milepost moments of the pandemic in the early-outbreak: Framework and applications to the COVID-19. F1000research.

[38] Cox J.D.L., Greenwald S.C.L. (2020). What explains the COVID-19 stock market?. NBER Work. Pap. Ser..

[39] Mandelbrot B. (1967). The variation of some other speculative prices. J. Bus..

[40] Engle R.F. (1982). Autoregressive conditional heteroskedasticity with estimates of the variance of UK. inflation. Econom. J. Econom. Soc..

[41] Bollerslev T., Chou R.Y., Kroner K.F. (1992). ARCH modeling in finance: A review of the theory and empirical evidence. J. Econ..

[42] Cheong C.W. (2009). Modeling and forecasting crude oil markets using ARCH-type models. Energy Policy.

[43] Bentes S.R., Menezes R., Mendes D.A. (2008). Long memory and volatility clustering: Is the empirical evidence consistent across stock markets?. Phys. A Stat. Mech. Appl..

[44] Ihlen E.A.F.E. (2012). Introduction to multifractal detrended fluctuation analysis in matlab. Front. Physiol..

[45] Wood A., Wood R., Charnley M. (2020). Through-the-wall radar detection using machine learning. Results Appl. Math..

[46] Manu K.S., Kalra R., Shubhika M. (2020). Stock index prediction using artificial neural network and econometric model: The case of nifty 50. Int. J. Adv. Sci. Technol..

